# Germline variants in cancer therapy

**DOI:** 10.20517/cdr.2019.05

**Published:** 2019-03-19

**Authors:** Meike Kaehler, Ingolf Cascorbi

**Affiliations:** Institute for Experimental and Clinical Pharmacology, University Hospital Schleswig-Holstein, Campus Kiel, Kiel D-24105, Germany.

**Keywords:** Pharmacogenetics, cytotoxic drugs, anticancer drugs, toxicity, drug resistance, drug metabolism, drug transporter

## Abstract

Cancer pharmacogenetics implies a complex combination of germline variants from the patient and somatic mutations in tumor cells. Somatic mutations meanwhile have become drugable targets or biomarkers, whereas germline mutations potentially predict adverse drug effects or drug response. Here, we evaluate hereditary variants in biotransforming enzymes and drug transporters, such as thiopurine S-methyltransferase, UDP-glucuronosyltransferase (*UGT1A1*), dihydropyrimidine dehydrogenase (*DPD*), as well as ABC transporters (*ABCB1*, *ABCG2* and *ABCC* subfamily) with respect to cytostatics and targeted therapies. Furthermore, gene expression regulation with regards to epigenetics and posttranscriptional modification are discussed.

## Introduction

The tumor cell genome consists of a complex combination of germline and somatic mutations that potentially interfere with anticancer treatment^[1]^. Most genetic mutations in a tumor cell are somatic and are increasingly implicated in targeted therapy with considerable therapeutic benefit. Nevertheless, germline variants may also contribute to interindividual differences in anti-cancer drug response leading to drug resistance, but also to adverse drug effects. Thus, somatic mutations are often associated with treatment efficacy, while hereditary variants are more often considered to address adverse drug effects^[2,3]^. As anticancer treatment often is a double-edged sword balancing best treatment outcome and potential life-threatening adverse drug effects, pharmacogenetic data is relevant to improve individualized therapy. In this context, variants in biotransforming enzymes, drug transporters and their regulators are of major interest as potential biomarkers for improvement of treatment regimen. Here, we discuss the impact of pharmacogenetic testing in treatment with cytostatics and targeted therapies in the context of drug resistance.

## Cytostatics, targeted therapy and beyond

Due to the lack of specific drug targets in various types of cancer and beneficial effects in combination therapy, cytostatics are still of major relevance for anticancer treatment. Metabolism and biotransformation of these compounds underlie various enzymes and transporters, which are well described^[4]^. Hence, hereditary variants in biotransforming enzymes are well known to impair metabolism of drugs, e.g., 5-fluorouracil (5-FU) or 6-mercaptopurine (6-MP). For several cytostatic compounds, pharmacogenetic information has been integrated into treatment recommendations of the Federal Drug Administration or guidelines from the Clinical Pharmacogenetics Implementation Consortium (CPIC), e.g., for 5-FU, irinotecan or 6-MP^[5]^. However, some pharmacogenetic markers still have not been translated into clinical practice.

Within the last 15 years, the number of targeted therapies using small molecules and antibodies drastically expanded with good clinical outcome and increased patient survival. One of the first compounds used was trastuzumab, a HER2/neu-antibody for the use in HER2+ breast cancer^[6]^ and the tyrosine kinase inhibitor imatinib, which targets the BCR/ABL1-kinase in chronic myeloid leukemia^[7]^. In contrast to classical chemotherapeutics, these substances rely on the presence or overexpression of drugable targets on/in the tumor cells. Therefore, expression of the respective target protein in the cancer cell is mandatory and genetic testing of cancer patients is compulsory, e.g., for HER2/neu overexpression for the use of trastuzumab, HER1 for the use of cetuximab or panitumumab or BCR/ABL1/c-kit for the use of imatinib. All of these compounds are dependent on binding to their target protein, which can be impaired by mutation or amplification and by this trigger chemoresistance. While this is a concern in treatment with antibodies and small molecules, drug level of the latter can also be impaired by several other mechanisms. Moreover, intracellular level can be reduced by ATP-binding cassette (ABC) transporters, which facilitate drug efflux or diminished expression or activity of drug importers. Some small molecules are substrates for the CYP450 enzymes, by which the drug plasma level could be impaired. These mechanisms leading to impaired drug level and transport are described below.

## Hereditary variants in biotransforming enzymes

### Purine analogues, TPMT and NUDT15

The association of thiopurine *S*-methyltransferase (TPMT) and 6-MP is one of the best documented pharmacogenetic interactions. This purine analogue, as well as azathioprine or thioguanine, are of major relevance in therapy of acute lymphoblastic leukemia (ALL) or non-malignant diseases, e.g., inflammatory bowel disease^[8-10]^. Its anti-inflammatory and anticancer mechanism is based on the generation of active, cytotoxic 6-thioguanosines having major negative impact on leucocyte proliferation. TPMT however prevents the organism from the formation of large amounts of these toxic intermediates through methylation and conversion to inactive methylmercaptopurine. Therefore, patients with TPMT deficiency suffer from a high risk of cytotoxicity due to accumulation of thioguanine nucleotides (6-TGN). Complete absence of TPMT activity may lead to myelosuppression and pancytopenia [Table 1]^[11]^.

**Table 1:** Association of hereditary variants in ADME genes to drug-induced adverse effects or therapy response

Therapeutics	Drug	Phase I		Phase II		Efflux transporters			Others
		*CYP2D6*	*TPMT*	*DPYD*	*UGT1A1*	*ABCB1*	*ABCG2*	*ABCC family*	
				DPYD*2A	UGT1A1*28	rs1045642, rs1128503, rs2032582	rs2231142, rs2231137, rs72552713	rs7177620, rs8187710	
Purine analogues	6-Mercaptopurine Azathioprine Thioguanine	-	+++ Myelosupression	-	-	-	-	-	+++ *NUDT15* R139C
Topoisomerase inhibitors	Irinotecan	-	-	-	++ Toxicity	-	(+)	-	-
Pyrimidin analogues	5-Fluorouracil Capecitabin	-	-	++ Toxicity	-	-	-	-	-
Hormone receptor antagonists	Tamoxifen	++ Response	-	-	-	-	-	-	-
Antimetabolites	Methotrexate	-	-	-	-	(+)		+	-
Tyrosine kinase inhibitors	Imatinib Sunitinib Erlotinib	-	-	-	-	(+)	(+)	-	-
Platinum derivates	Carboplatin Cisplatin	-	-	-	-	- +	- +	(+)	-
Anthracyclines	Doxorubicin	-	-	-	-	-	-	+ Cardiotoxicity	*+* *UGT1A6*,*RARγ, SLC28A3*
Vinca alkaloids	Vincristin	-	-	-	-	+	+	-	*-*
Enzymes	L-Asparaginase	-	-	-	-	-	-	-	*+* *GRIA1*

+++: very strong evidence; ++: strong evidence; +: weak evidence; (+): conflicting data, further studies necessary; -: lack of association [classification based on CPID guidelines (+++,++) and literature databases (+, (+))]

The frequency of 6-TGN-related hematologic adverse drug events depend on the *TPMT* genotype, while intestinal side effects seem to be genotype-independent^[12]^. Nevertheless, pharmacogenetics testing and subsequent adjusting the purine analogue dose is highly recommended^[13]^.

Only three SNPs, namely *3A, *3C and *2, account for more than 90% of TPMT inactivating variants in the Caucasian population^[13,14]^. According to the *TPMT* genotype, the phenotype is distinguished into normal, intermediate and poor metabolizers. Some diplotypes are still considered as “undetermined” such as TPMT*1/*8 or *6/*8. The most recent CPIC guideline suggests starting dose reductions of 50%-80% for immediate, while the starting dose in poor metabolizers should be drastically reduced to 10% and administered thrice weekly instead of daily^[23]^. The dose should be carefully titrated depending on the grade of myelosuppression^[15]^.

Interestingly, TPMT seems to be of particular relevance in Caucasian, while in Asian population *TPMT* variants are less frequent, but the degree of leukopenia is more pronounced^[16,17]^. In Asian populations, another polymorphic gene was discovered to play an important role in thiopurine toxicity. Yang and co-workers identified a significant association of the NUDT15 (nucleoside diphosphate-linked moiety X type motif 15) variant p.R139C to thiopurine-related leukopenia, which is involved in nucleoside diphosphate metabolism, especially built in stress response^[18]^. In this study among children with ALL, those being homozygote for the variant allele p.R139C tolerated only 8% of the standard mercaptopurine dose. In contrast, the tolerated dose intensity in heterozygous and wildtype carriers was 63% and 83.5%, respectively. As NUDT15 catalyzes thiopurine inactivation to thioguanine monophosphate, the almost complete loss of NUDT15 activity led to accumulation of thiopurine-metabolites causing toxicity [Table 1]^[19-21]^. Meanwhile further variant alleles have been identified with varying prevalence among different ethnicities and varying and sometimes uncertain functional effects. A recommendation of a *NUDT15* nomenclature was published very recently by the Pharmacogene Variation (PharmVar) Consortium^[22]^.

Due to the high clinical significance of *NUDT15* gene variation especially for Asian populations, the CPIC guideline for thiopurine dosing was amended^[23]^. To avoid thiopurine toxicity both, *TPMT* as well as *NUDT15* genotypes should be considered, although the *NUDT15* genotype-related dose recommendations are not yet fully clear.

### Irinotecan and UGT1A1

*N*- or *O*-glucuronidation by specific UDP-glucoronosyltransferases (UGT) is a crucial step in the elimination process of a number of drugs and endogenous compounds. Usually such conjugation elevates the compound’s hydrophilicity thereby facilitating excretion into urine. This is exemplified by UGT1A1 that catalyzes the transformation of bilirubin to bilirubin-glucuronide in the liver, before this conjugate can be secreted via the renal ABC transporter ABCC2 into urine.

In this context, UGT1A1*28 (rs8175347), a TA tandem repeat in the *UGT1A1* promoter causing Gilbert syndrome/Morbus Meulengracht is of relevance causing defective heme metabolism. Wild type alleles harbor six TA repeats, whereas the seven TA repeat variant leads to diminished protein expression. Although presence of UGT1A1*28 is abundant in Caucasians (minor allele frequency of carriers 40%), this genetic variant itself does not contribute to development of defective heme metabolism, as frequency of Gilbert syndrome is only 3%-9% in Europeans^[24-27]^.

The topoisomerase I-inhibitor irinotecan, widely used for the treatment of colorectal cancer in combination with 5-FU, is one of the drugs being metabolized by UGT1A1. Carriers of UGT1A1*28 variants bear a higher risk for drug toxicities as UGT1A1 is required for detoxification of the drug. Low UGT1A1 activity may result in diarrhea and neutropenia in a dose-dependent manner [Table 1]^[28]^. So far, UGT1A1*28 genotyping is recommended as part of the irinotecan drug label, although no distinct doses are given. Dependent on the dosage, patients being heterozygous carriers should be closely monitored for any toxicities, while for homozygous carriers the starting dose should be reduced^[15,29]^. Recently, a study in Japanese colorectal cancer patients revealed that reduction of the initial irinotecan dose from 150 mg/m² to 120 mg/m² results in a safe and efficient therapy in *UGT1A1* homozygote variant allele carriers^[30]^. It should be noted that among Asian populations, c.211G>A leading to p.Gly71Arg and allocated to UGT1A1*6 should be considered as well due the low activity phenotype. A retrospective study from Switzerland however could not confirm that UGT1A1*28 is the only risk factor for neutropenia and diarrhea. In a multifactorial analysis baseline neutrophil count, sex, age and performance status were additional items contributing to the complexity of adverse events^[31]^. Studies investigating the question, whether elevated irinotecan doses are tolerable among wild-type carriers are ongoing. Here, pharmacogenetic testing could contribute not only to the avoidance of toxicity, but also to a putative better clinical outcome of the disease.

### Pyrimidine analogues and dihydropyrimide dehydrogenase

The pyrimidine analogue 5-FU is commonly used for treatment of metastatic colon carcinoma or breast cancer. Main metabolism of the drug is performed in the liver by dihydropyrimide dehydrogenases (DPD), which also metabolize other fluoropyrimidines, e.g., capecitabin. Defects in DPD lead to accumulation of 5-FU and systemic toxicity, ranging from myelosuppression, neurotoxicity or diarrhea [Table 1].

For DPD, several rare variants have been described (also reviewed in^[32]^): DPYD*2A (rs3918290) leads to the formation of an alternate splicing side in intron 14, resulting in expression of a 165 bp-deleted *DPYD* mRNA and protein lacking the amino acid residues 581-635^[33]^. The AA variants leads to complete DPD deficiency and severe 5-FU toxicities^[34]^. Especially in African-Americans, DPYD*9A (rs1801265) has been described in 13%-39% of the population^[35]^. The effect of this variant is still controversial, as some studies revealed association of DPYD*9A genotype and 5-FU side effects in gastrointestinal malignancies^[36]^, but others did not observe impairment of enzyme activity of the 9A haplotype^[37]^. Overall, this points to a dependency of this haplotype to other non-genetic factors.

For further rare genetic variants with low frequencies, especially DPYD*3 (rs72549303) and DPYD*4 (rs1801158), analyses of effects and dose-adjustments is still ongoing. In an *in vitro*-expression system, reduced enzyme activity was observed for D949V, while M166V, E828K, K861R and P1023T variants leading to increased enzyme activity compared to the wild type^[35,38]^.

DPD activity scores are based on the functionally characterized variants c.190511G>A, c.1679T>G, c.2846A>T, and. c.1129-5923C>G. Based on these estimated activity scores, the recent CPIC guidelines strongly suggest for patients with complete DPD deficiency to avoid the use of fluoropyrimidines^[39]^. This is also due for patients having a very low activity score of 0.5. In cases were no alternative for 5-FU are considered, strong dose reduction and therapeutic drug monitoring is recommended. Since also intermediate metabolizers have an increased risk of adverse events, dose reductions of 25%-50% should be considered.

Moreover, combinational analysis of *DPYD* and *UGT1A1* genotypes in Italian colorectal cancer patients receiving fluoropyrimidines/irinotecan revealed that the incremental cost between *DPYD* variant and UGT1A1*28/*28 carriers and non-carriers was €2,975, indicating the relevance of these pharmacogenetic traits also from an economic perspective^[40]^. In addition, a recent study stressed the relevance of *DPYD* testing accompanied by genotype-dependent dose reduction for the use of fluoropyrimidines^[41]^. Concluding, patients not only of non-European descent could benefit from genetic testing for *DPYD/UGT1A1* variants.

### Tamoxifen and CYP2D6

The estrogen receptor antagonist tamoxifen has been successfully used in treatment of estrogen-receptor positive breast cancer since more than 40 years. Tamoxifen is metabolized mainly by CYP2D6 resulting in various metabolites of which endoxifen has the strongest affinity to the estrogen receptor. As a result, *CYP2D6* genotype tightly correlates to endoxifen plasma levels in patients. In several studies it was shown that *CYP2D6* genotype is a determinant marker of tamoxifen efficacy [Table 1] contributing to 34%-52 % of absolute endoxifen plasma level^[42]^. Several variants have been described for *CYP2D6* being associated with normal (CYP2D6*1, CYP2D6*2), decreased (e.g., CYP2D6*10) or complete loss of function (e.g., CYP2D6*3, CYP2D6*4). Furthermore, *CYP2D6* copy number variants may lead to excessive metabolism and ultrarapid CYP2D6 phenotype (reviewed in^[43]^). Indeed, several studies suggested an association of *CYP2D6* genotype and overall survival, relapse- or recurrence-free survival^[43-46]^. Accordingly, the CPIC guidelines for *CYP2D6* and tamoxifen therapy recommend that CYP2D6 poor metabolizers should be treated alternatively with aromatase inhibitors and in CYP2D6 intermediates dosage adjustments should be performed to prevent therapy failure of tamoxifen. In addition, CYP2D6 inhibitors should be avoided regardless of the *CYP2D6* genotype^[43]^. Regarding the use of treatment alternatives, esp. aromatase inhibitors, their use in pre-menopausal patient still is contraindicated due to occurrence of treatment-associated amenorrhea, unless suppression of ovarian function is performed simultaneously^[47]^. This has been shown in recent studies, clearly showing superior overall and disease-free survival rates in combination of aromatase inhibitors or tamoxifen plus ovarian suppression^[48]^. In post-menopausal patients, the optimal treatment strategy is still controversial, as treatment-duration and -switching between tamoxifen and aromatase inhibitors is still under investigation^[49,50]^. This adds additional complexity to the relevance of *CYP2D6* genotyping for the use of tamoxifen. So far, due to a number of inconsistencies, pharmacogenetic analysis of *CYP2D6* status has not yet been considered in the drug label. As reviewed recently, some inconsistencies in the role of *CYP2D6* can be explained by the proportion of DNA isolated from peripheral blood cells or tumor tissue. So far, any association of treatment outcome to *CYP2D6* genotype failed when using tumor tissue as DNA source^[51]^. However, in 960 women from the Quilt Study cohort, neither the *CYP2D6* genotype nor the concomitant use of CYP2D6 inhibitors had any influence on the clinical outcome^[52]^. In addition, in a prospective study including 667 pre- and postmenopausal patients, no association was found between endoxifen concentrations and relapse-free survival time, and there was also lack of association between *CYP2D6* genotype and relapse-free survival time. As any selection bias or non-consideration of confounding factors was regarded as small, the authors concluded, that the results of this study do not support *CYP2D6* genotyping to guide tamoxifen treatment in the adjuvant setting^[53]^.

### Promising variants as new predictive factors

Within the last years, several studies revealed novel promising candidates hereditary variants apart from ADME genes that might help to predict ADE under chemotherapy regimen. For these, we would like to stress some recent findings in anthracycline-derived cardiotoxicity and *L*-asparaginase-induced hypersensitivity reactions.

Anthracylines, e.g., doxorubicin or daunorubicin, are of major relevance for the treatment of multiple solid tumors and leukemia. Severe side effects, in particular cardiotoxicity occurring in about 57% of all patients is a frequent dose limiting cause. In the last years, several studies have shown hereditary variants in UDP-glucuronosyltransferase 1A6 (*UGT1A6*; taq marker of UGT1A6*4, rs17863783), the nucleoside transporter *SLC28A3* (L461L, rs7853758; intronic variant, rs885004) or retinoic acid receptor gamma (*RAR*γ; rs2229774) as promising predictive biomarkers (comprehensively reviewed in^[54]^). The silent variant V209V (rs17863783) in *UGT1A6* was associated with increased risk of anthracycline-induced cardiotoxicity in several patient cohorts, potentially resulting in decrease of enzyme function and accumulation of toxic metabolites^[55,56]^. A similar phenomena has been shown for the sodium-coupled nucleoside transporter *SLC28A3*, as rs7853758 and rs885004 might interfere with enzyme activity promoting risk of cardiotoxicity^[57]^. In addition, the amino acid exchange S427L (rs2229774) in retinoic acid receptor gamma (*RAR*γ) was found in a GWAS study to be associated with cardiotoxic risk increase in children^[58]^. Earlier studies reported an association of *ABCC1* efflux transporter variants with doxorubicin-associated cardiomyopathy^[59]^. However, so far the predictive value of these variants is not determined or low, preventing its implementation into clinical practice so far.

Regarding the use of *L*-asparaginase in ALL, hypersensitivity reaction have been observed. Interestingly, pharmacogenetics studies revealed an association to multiple polymorphisms (rs4958351, rs10070447, rs6890057, rs4958676, rs6889909, rs11167640, rs10072570, rs13354399, rs17356099, rs2055083, rs707176) in the AMPA 1 glutamate receptor (GRIA1)^[60,61]^. These data clearly point to inherited factors contributing to treatment hypersensitivity, however, functional validation is necessary to understand the relevance of these variants.

Finally, the treatment of solid tumors with taxanes, such as paclitaxel, docetaxel, and cabazitaxel is commonly associated with treatment-limiting peripheral sensory neuropathy. Currently there are no biomarkers available, allowing a precise prediction of such chemotherapy-induced peripheral neuropathy (CIPN)^[62]^. The attempt to reveal predictive genetic markers through GWAS studies disclosed an intronic SNP (rs875858, MAF ¼ 0.056), in the *VAC14* gene. VAC14 encodes a scaffold protein that is a component of the PIKfyve protein kinase complex. Interestingly this complex is involved in the synthesis of phosphatidylinositol 3,5-bisphosphate. Knock-out experiments in mice caused severe neurodegeneration^[63]^. The authors of the GWAS study further made mechanistic approaches to validate the functional consequences of the *VAC14* variant. iRNA knockdown of *VAC14* in stem cell-derived peripheral neuronal cells increased docetaxel sensitivity and *VAC14* heterozygous mice had greater nociceptive sensitivity than wild-type controls. Further GWAS meta-analyses of taxane-related CIPN were however not able to identify significant associations of any genetic variant to ≥ grade common terminology criteria for adverse events (CTCAE) neuropathy in European and African Americans after correction for multiple testing^[64]^. Concluding, there are some interesting genetic risk candidates, however their suitability for predicting treatment-limiting peripheral sensory neuropathy has not been confirmed so far.

## Multidrug resistance in cancer treatment and its pharmacogenetic contributions

The term “multidrug resistance” is tightly linked to expression of ABC-transporters in cancer cells^[65]^. By efflux of substrates across the membrane, the intracellular level of the drug is lowered and drug resistance becomes more likely. Furthermore, bioavailability of drugs is impaired influencing adsorption, distribution and elimination. The most prominent transporters regarding chemoresistance are P-glycoprotein (P-gp, ABCB1), breast cancer resistance protein (BCRP, ABCG2) and members of the ABCC-family, especially ABCC1/MRP1 (multidrug resistance protein 1), ABCC2/MRP2 (multidrug resistance protein 2) and ABCC3. Drug resistance via drug transporters is not only influenced by deregulation of gene expression, but also by genetic variants^[66]^. While a vast number of studies showed impact of genetic variants on drug transporter expression or activities, recent data dealt with variation of 3’-UTR length in drug resistant cancer cells.

### P-gp in drug resistance

A huge number of classical and targeted chemotherapeutics are substrates for P-gp and many studies demonstrated an overexpression of P-gp upon exposure to such drugs [Table 1]. Consequently, a potential impact of genetic variants on *ABCB1* expression and subsequently in response to treatment could be found with more than 4,453 SNPs identified in this gene. Most clinical studies focused on 3435C>T, 2677G>T/A and 1236C>T that were suggested to impair P-gp expression or activity.

The cytosine deamination at position 3435 (rs1045642) in exon 26 does not result in an amino acid substitution. Nevertheless, a number of studies suggested a lower P-gp expression and reduced P-gp function in 3435T carriers^[67,68]^. In acute lymphocytic leukemia, carriers of the wildtype revealed higher toxicity after high-dose methotrexate treatment, while the risk of relapse was reduced in carriers of at least one variant allele^[69]^. Nonetheless, a direct association to cancer therapy failure could not be seen. The impact of the silent SNP 1236C>T (rs1128503) in exon 12 instead is not entirely understood, as contradictory studies showed opposite results regarding response to imatinib in chronic myeloid leukemia^[70,71]^. In the same studies, amino acid substitution of alanine to serine/threonine due to 2677G>T/A (A893T, rs2032582) could be associated to imatinib response, with contradictory effects as carriers of the CC variant revealed altered susceptibility towards imatinib therapy compared to wildtype carriers. Regarding missense mutations in *ABCB1*, an experimental approach using *Saccharomyces*-based assay revealed several non-synonymous SNPs in the context of chemoresistance, e.g., 266T>C (M89T), 1985T>G (L662R), 2005C>T (R669C) 3322T>C (W1108R) and 3421T>A (S1141T)^[72,73]^. Nevertheless, clinical relevance of these SNPs has to be confirmed. Overall, the issue on *ABCB1* variants and their association to drug response remains controversial. Due to its high phenotypic variability however, it is more than questionable to use *ABCB1* variants to as predictive biomarker in cancer therapy. In addition to hereditary variants, the regulation of ABC transporters by nuclear receptors, cytokines and also microRNAs is of major importance. In this regard, differential expression of 3’-UTR lengths of the *ABCB1* mRNA might additionally contribute to P-gp variability (see epigenetics section).

### ABCG2/BCRP and chemoresistance

Besides P-gp, BCRP was identified as a contributing factor to multidrug resistance [Table 1]. Being highly expressed in hematopoetic precursor and stem cells^[74]^, it is regarded as a stem cell factor^[75]^. Moreover, its high expression in tumor cells pointed to a potential role in chemoresistance and therapy failure^[76,77]^. Main research has been performed on non-synonymous 34G>A and 421C>A. The SNP 34G>A (rs2231137) results in exchange of valine to methionine (V12M) at the N-terminus potentially leading to reduced protein expression. This SNP could be associated to outcome of tyrosine kinase inhibitor therapy, especially using imatinib^[78]^. Some clinical evidence point to a better response to imatinib in CML patients carrying the homozygous variant^[78]^. A similar tendency was observed in metastatic renal cell cancer treated with sunitinib^[79]^. On the contrary, an association of therapy using erlotinib in B cell lymphoma patients could not be observed^[80]^. The 421C>A substitution (rs2231142) leads to an amino acid exchange from glutamine to lysine (Q141K) that affects the ATP binding domain. In a number of studies, bioavailability of drugs, e.g., the topoisomerase inhibitor topotecan^[81]^ or the tyrosine kinase inhibitor imatinib^[82]^, was impaired in carriers of the 421C>A genotype. Regarding tyrosine kinase inhibitors, many compounds are substrates of BCRP, however transport is dose-dependent^[83]^. Interestingly, the 421A haplotype could be associated to modulate posttranscriptional regulation via the 3’-UTR^[84]^. Nevertheless, no significant association of 421C>A was found or results were contradictory. Besides these two SNPs, 376C>T leads to formation of a premature stop codon (Q126stop, rs72552713) resulting in diminished protein expression and could be associated to drug hypersensitivity^[85]^. Conversely, the promoter SNP -15994C>T results in increased BCRP expression and imatinib clearance^[86]^.

### The ABCC subfamily and their genetic variants in chemoresistance

For the ABCC subfamily, twelve members have been described yet. With regards to chemoresistance ABCC1, ABCC2 and ABCC3 have been intensively investigated. While anticancer drugs of the new generation are mainly transported by ABCB1 and ABCG2, several cytostatics, e.g., vincristine or cisplatin, are substrates for these transporters [Table 1]^[87]^. As distinct clinical studies on association of ABCC1 and ABCC3 to drug resistance are lacking, most data is present on ABCC2. Most prominent SNPs are -24C>T in the *ABCC2* promoter region (rs7177620) being associated to reduced expression and activity of the protein^[68]^. This could thus be linked to higher methotrexate plasma levels^[88]^. Together with 1249G and 3972T, -24T haplotype was associated with imatinib resistance^[89]^. Regarding therapy using platinum or platinum-derivates, e.g., carboplatin, it was observed that 4544G>A (rs8187710) correlated with adverse survival rates of patients suffering from non-small cell lung cancer^[90]^. Furthermore, this SNP was associated with doxorubicin-induced cardiotoxicity requiring closer monitoring during treatment^[59]^.

### Chemoresistance, epigenetics and miRNA regulation

It is well known that protein expression can be modulated on the transcriptional level by epigenetics, as well as post-transcriptionally by microRNAs or RNA-binding proteins. Analyses of epigenetics, e.g., DNA methylation or histone modification, remain inconclusive for ADME genes so far. In several studies, data on promoter methylation of the efflux transporters *ABCB1* or *ABCG2* showed contrary differential methylation in various types of cancer^[91-96]^. In addition, hereditary variants in promoter regions might directly impair binding of transcription factors leading to differential gene expression. This has also been shown for several cytochrome P450 enzymes pointing to gene expression regulation by methylation, e.g., CYP1A2, CYP2C19 and CYP2D6^[97,98]^.

Main research has been performed for transcriptional control by microRNAs resulting in blockade of translation or degradation of their respective target mRNAs^[99]^. This interaction can be impaired by hereditary variants of the mRNA 3’-UTRs in target genes. For ABC-transporters, several microRNA interactions have been described so far, e.g., miR-508-3p/*ABCB1*, miR-212/*ABCG2* or miR-379/*ABCC2*^[91,100,101]^. For the latter, it was observed that *ABCC2* -24C>T, 1249G>A and 3972C>T variant haplotypes led to pronounced microRNA-dependent suppression compared to wild-types^[102]^.

Interestingly, the interaction of microRNA/mRNA can also be impaired by selective expression of alternative mRNA 3’-UTRs by the cancer cell. As these shorter 3’-UTRs lack the microRNA-binding regions, regulation of respective microRNA can be abolished. This phenomenon was observed not only for *ABCG2* in mitoxantrone-resistant colon cancer cells, but also for *ABCB1* in chronic myeloid leukemia cells^[103,104]^. Potentially, this is also relevant for other ABC transporters or biotransforming enzymes (reviewed in^[105,106]^). Further studies are required to investigate this mechanism and its regulation.

Within the last decade it was observed that microRNA expression pattern are distinct for each tumor and can be altered during in treatment with anticancer drugs (reviewed in^[107]^). Thus, haplotype-dependent microRNA interaction and regulation of mRNA expression might contribute to differential susceptibility of tumors and patients.

## Conclusions and outlook

Pharmacogenetic testing of somatic and germline mutations is an emerging field in oncology. Presence of hereditary variants are relevant for avoidance of adverse drug events in some classic chemotherapy regimen, such as thiopurines, 5-FU or irinotecan. Pharmacogenetic testing for hereditary variants in metabolic enzymes like *TPMT* and *NUDT15*, *UGT1A1* or *DPYD* might be helpful to prevent patients from severe adverse drug effects by subsequent adjustment of the drug dose or therapy alternatives. Although genetic testing for these traits is not mandatory, implementation into the respective drug label has been performed and genetic testing is recommended. Overall, testing for hereditary variants in these genes might be helpful to predict adverse drug effects and improve patients’ compliance.

In contrast to variants in biotransforming enzymes, association of SNPs in drug transporters to the clinical outcome is controversially discussed. Evidence on the predictive value of many SNPs on the respective gene expression, function and association to drug efficacy is widely lacking. Therefore, the impact on hereditary variants in ABC transporters on overall therapy response remains questionable.

Overall, pharmacogenetic testing of single traits might not be sufficient to explain neither interindividual differences, nor intratumoral differences in many cases, as these underlie clonal evolution. Within the last decades, there is much more understanding of tumor heterogeneity and complexity and research has been widely evolved. Technologies such as whole genome analyses, RNA-seq or epigenetic analyses have been shown to be crucial to gain deeper insights into the complexity of tumor traits and to design novel therapy strategies and co-treatments. Especially for targeted therapy regimen, testing for presence of drugable targets in or on tumor cells is mandatory (e.g., BCR/ABL1 or HER2). Moreover accompanied diagnostics of tumor mutations is required for an increasing number of anticancer drugs including biologicals. Furthermore, the invention of checkpoint blockade inhibitors like ipilimumab, nivolumab and pembrolizumab leading to re-activation of T cell anti-tumor response revolutionized treatment in a variety of malignancies^[108-110]^. For these compounds, tumor dependency on the respective target is necessary for therapeutic benefit.

Nevertheless, co-integration of genetic testing into the clinical routine for somatic and germline variants in cancer simultaneously could be helpful to optimize therapy regimen and improve patients’ compliance and survival.

## Declarations

### Copyright

© The Author(s) 2019.

## References

[1] Relling MV, Evans WE (2015). Pharmacogenomics in the clinic.. Nature.

[2] Filipski KK, Mechanic LE, Long R, Freedman AN (2014). Pharmacogenomics in oncology care.. Front Genet.

[3] Gillis NK, Patel JN, Innocenti F (2014). Clinical implementation of germ line cancer pharmacogenetic variants during the next-generation sequencing era.. Clin Pharmacol Ther.

[4] Oscarson M (2003). Pharmacogenetics of drug metabolising enzymes: importance for personalised medicine.. Clin Chem Lab Med.

[5] Caudle KE, Thorn CF, Klein TE, Swen JJ, McLeod HL (2013). Clinical pharmacogenetics implementation consortium guidelines for dihydropyrimidine dehydrogenase genotype and fluoropyrimidine dosing.. Clin Pharmacol Ther.

[6] Cameron D, Piccart-Gebhart MJ, Gelber RD, Procter M, Goldhirsch A (2017). 11 years’ follow-up of trastuzumab after adjuvant chemotherapy in HER2-positive early breast cancer: final analysis of the HERceptin adjuvant (HERA) trial.. Lancet.

[7] O’Dwyer ME, Druker BJ (2000). STI571: an inhibitor of the BCR-ABL tyrosine kinase for the treatment of chronic myelogenous leukaemia.. Lancet Oncol.

[8] Carr DF, Pirmohamed M (2018). Biomarkers of adverse drug reactions.. Exp Biol Med (Maywood).

[9] Abaji R, Krajinovic M (2017). Thiopurine S-methyltransferase polymorphisms in acute lymphoblastic leukemia, inflammatory bowel disease and autoimmune disorders: influence on treatment response.. Pharmgenomics Pers Med.

[10] Lee SHR, Yang JJ (2017). Pharmacogenomics in acute lymphoblastic leukemia.. Best Pract Res Clin Haematol.

[11] Paugh SW, Stocco G, McCorkle JR, Diouf B, Crews KR (2011). Cancer pharmacogenomics.. Clin Pharmacol Ther.

[12] Schwab M, Schaffeler E, Marx C, Fischer C, Lang T (2002). Azathioprine therapy and adverse drug reactions in patients with inflammatory bowel disease: impact of thiopurine S-methyltransferase polymorphism.. Pharmacogenetics.

[13] Relling MV, Gardner EE, Sandborn WJ, Schmiegelow K, Pui CH (2011). Clinical Pharmacogenetics Implementation Consortium guidelines for thiopurine methyltransferase genotype and thiopurine dosing.. Clin Pharmacol Ther.

[14] Schaeffeler E, Fischer C, Brockmeier D, Wernet D, Moerike K (2004). Comprehensive analysis of thiopurine S-methyltransferase phenotype-genotype correlation in a large population of German-Caucasians and identification of novel TPMT variants.. Pharmacogenetics.

[15] Wellmann R, Borden BA, Danahey K, Nanda R, Polite BN (2018). Analyzing the clinical actionability of germline pharmacogenomic findings in oncology.. Cancer.

[16] Kim JH, Cheon JH, Hong SS, Eun CS, Byeon JS (2010). Influences of thiopurine methyltransferase genotype and activity on thiopurine-induced leukopenia in Korean patients with inflammatory bowel disease: a retrospective cohort study.. J Clin Gastroenterol.

[17] Jung YS, Cheon JH, Park JJ, Moon CM, Kim ES (2010). Correlation of genotypes for thiopurine methyltransferase and inosine triphosphate pyrophosphatase with long-term clinical outcomes in Korean patients with inflammatory bowel diseases during treatment with thiopurine drugs.. J Hum Genet.

[18] Yang SK, Hong M, Baek J, Choi H, Zhao W (2014). A common missense variant in NUDT15 confers susceptibility to thiopurine-induced leukopenia.. Nat Genet.

[19] Moriyama T, Nishii R, Lin TN, Kihira K, Toyoda H (2017). The effects of inherited NUDT15 polymorphisms on thiopurine active metabolites in Japanese children with acute lymphoblastic leukemia.. Pharmacogenet Genomics.

[20] Moriyama T, Yang YL, Nishii R, Ariffin H, Liu C (2017). Novel variants in NUDT15 and thiopurine intolerance in children with acute lymphoblastic leukemia from diverse ancestry.. Blood.

[21] Yang JJ, Landier W, Yang W, Liu C, Hageman L (2015). Inherited NUDT15 variant is a genetic determinant of mercaptopurine intolerance in children with acute lymphoblastic leukemia.. J Clin Oncol.

[22] Yang JJ, Whirl-Carrillo M, Scott SA, Turner AJ, Schwab M (2018). Pharmacogene variation consortium gene introduction: NUDT15.. Clin Pharmacol Ther.

[23] Relling MV, Schwab M, Whirl-Carrillo M, Suarez-Kurtz G, Pui CH (2018). Clinical pharmacogenetics implementation consortium (CPIC) guideline for thiopurine dosing based on TPMT and NUDT15 genotypes: 2018 update.. Clin Pharmacol Ther.

[24] Bosma P, Chowdhury JR, Jansen PH (1995). Genetic inheritance of Gilbert’s syndrome.. Lancet.

[25] Bosma PJ, Chowdhury JR, Bakker C, Gantla S, de Boer A (1995). The genetic basis of the reduced expression of bilirubin UDP-glucuronosyltransferase 1 in Gilbert’s syndrome.. N Engl J Med.

[26] Dean L (2012). Irinotecan therapy and UGT1A1 genotype. Medical Genetics Summaries..

[27] Barbarino JM, Haidar CE, Klein TE, Altman RB (2014). PharmGKB summary: very important pharmacogene information for UGT1A1.. Pharmacogenet Genomics.

[28] Hoskins JM, Goldberg RM, Qu P, Ibrahim JG, McLeod HL (2007). UGT1A1*28 genotype and irinotecan-induced neutropenia: dose matters.. J Natl Cancer Inst.

[29] Etienne-Grimaldi MC, Boyer JC, Thomas F, Quaranta S, Picard N (2015). UGT1A1 genotype and irinotecan therapy: general review and implementation in routine practice.. Fundam Clin Pharmacol.

[30] Fujii H, Yamada Y, Watanabe D, Matsuhashi N, Takahashi T (2019). Dose adjustment of irinotecan based on UGT1A1 polymorphisms in patients with colorectal cancer.. Cancer Chemother Pharmacol.

[31] Tejpar S, Yan P, Piessevaux H, Dietrich D, Brauchli P (2018). Clinical and pharmacogenetic determinants of 5-fluorouracyl/leucovorin/irinotecan toxicity: results of the PETACC-3 trial.. Eur J Cancer.

[32] Cecchin E, De Mattia E, Ecca F, Toffoli G (2018). Host genetic profiling to increase drug safety in colorectal cancer from discovery to implementation.. Drug Resist Updat.

[33] Meinsma R, Fernandez-Salguero P, Van Kuilenburg AB, Van Gennip AH, Gonzalez FJ (1995). Human polymorphism in drug metabolism: mutation in the dihydropyrimidine dehydrogenase gene results in exon skipping and thymine uracilurea.. DNA Cell Biol.

[34] Saif MW, Ezzeldin H, Vance K, Sellers S, Diasio RB (2007). DPYD*2A mutation: the most common mutation associated with DPD deficiency.. Cancer Chemother Pharmacol.

[35] Offer SM, Lee AM, Mattison LK, Fossum C, Wegner NJ (2013). A DPYD variant (Y186C) in individuals of african ancestry is associated with reduced DPD enzyme activity.. Clin Pharmacol Ther.

[36] Khushman M, Patel GK, Hosein PJ, Laurini JA, Cameron D (2018). Germline pharmacogenomics of DPYD*9A (c.85T>C) variant in patients with gastrointestinal malignancies treated with fluoropyrimidines.. J Gastrointest Oncol.

[37] Morel A, Boisdron-Celle M, Fey L, Soulie P, Craipeau MC (2006). Clinical relevance of different dihydropyrimidine dehydrogenase gene single nucleotide polymorphisms on 5-fluorouracil tolerance.. Mol Cancer Ther.

[38] Offer SM, Wegner NJ, Fossum C, Wang K, Diasio RB (2013). Phenotypic profiling of DPYD variations relevant to 5-fluorouracil sensitivity using real-time cellular analysis and in vitro measurement of enzyme activity.. Cancer Res.

[39] Amstutz U, Henricks LM, Offer SM, Barbarino J, Schellens JHM (2018). Clinical Pharmacogenetics Implementation Consortium (CPIC) Guideline for Dihydropyrimidine Dehydrogenase Genotype and Fluoropyrimidine Dosing: 2017 Update.. Clin Pharmacol Ther.

[40] Toffoli G, Innocenti F, Polesel J, De Mattia E, Sartor F (2018). The genotype for DPYD risk variants in patients with colorectal cancer and the related toxicity management costs in clinical practice.. Clin Pharmacol Ther.

[41] Henricks LM, Lunenburg C, de Man FM, Meulendijks D, Frederix GWJ (2018). DPYD genotype-guided dose individualisation of fluoropyrimidine therapy in patients with cancer: a prospective safety analysis.. Lancet Oncol.

[42] Schroth W, Antoniadou L, Fritz P, Schwab M, Muerdter T (2007). Breast cancer treatment outcome with adjuvant tamoxifen relative to patient CYP2D6 and CYP2C19 genotypes.. J Clin Oncol.

[43] Goetz MP, Sangkuhl K, Guchelaar HJ, Schwab M, Province M (2018). Clinical pharmacogenetics implementation consortium (CPIC) guideline for CYP2D6 and tamoxifen therapy.. Clin Pharmacol Ther.

[44] Brauch H, Schroth W, Goetz MP, Murdter TE, Winter S (2013). Tamoxifen use in postmenopausal breast cancer: CYP2D6 matters.. J Clin Oncol.

[45] Li J, Czene K, Brauch H, Schroth W, Saladores P (2013). Association of CYP2D6 metabolizer status with mammographic density change in response to tamoxifen treatment.. Breast Cancer Res.

[46] Province MA, Goetz MP, Brauch H, Flockhart DA, Hebert JM (2014). CYP2D6 genotype and adjuvant tamoxifen: meta-analysis of heterogeneous study populations.. Clin Pharmacol Ther.

[47] Pelizzari G, Arpino G, Biganzoli L, Cinieri S, De Laurentiis M (2018). An Italian delphi study to evaluate consensus on adjuvant endocrine therapy in premenopausal patients with breast cancer: the ERA project.. BMC Cancer.

[48] Francis PA, Pagani O, Fleming GF, Walley BA, Colleoni M (2018). Tailoring adjuvant endocrine therapy for premenopausal breast cancer.. N Engl J Med.

[49] Fabian CJ (2007). The what, why and how of aromatase inhibitors: hormonal agents for treatment and prevention of breast cancer.. Int J Clin Pract.

[50] Morales L, Neven P, Paridaens R (2005). Choosing between an aromatase inhibitor and tamoxifen in the adjuvant setting.. Curr Opin Oncol.

[51] Drogemoller BI, Wright GEB, Shih J, Monzon JG, Gelmon KA (2018). CYP2D6 as a treatment decision aid for ER-positive non-metastatic breast cancer patients: a systematic review with accompanying clinical practice guidelines.. Breast Cancer Res Treat.

[52] Mayer SE, Weiss NS, Chubak J, Doody DR, Carlson CS (2019). CYP2D6-inhibiting medication use and inherited CYP2D6 variation in relation to adverse breast cancer outcomes after tamoxifen therapy.. Cancer Causes Control.

[53] Sanchez-Spitman A, Dezentje V, Swen J, Moes D, Bohringer S (2019). Tamoxifen pharmacogenetics and metabolism: results from the prospective CYPTAM study.. J Clin Oncol.

[54] Aminkeng F, Ross CJ, Rassekh SR, Hwang S, Rieder MJ (2016). Recommendations for genetic testing to reduce the incidence of anthracycline-induced cardiotoxicity.. Br J Clin Pharmacol.

[55] Nagar S, Zalatoris JJ, Blanchard RL (2004). Human UGT1A6 pharmacogenetics: identification of a novel SNP, characterization of allele frequencies and functional analysis of recombinant allozymes in human liver tissue and in cultured cells.. Pharmacogenetics.

[56] Krishnaswamy S, Hao Q, Al-Rohaimi A, Hesse LM, von Moltke LL (2005). UDP glucuronosyltransferase (UGT) 1A6 pharmacogenetics: II. Functional impact of the three most common nonsynonymous UGT1A6 polymorphisms (S7A, T181A, and R184S).. J Pharmacol Exp Ther.

[57] Visscher H, Ross CJ, Rassekh SR, Sandor GS, Caron HN (2013). Validation of variants in SLC28A3 and UGT1A6 as genetic markers predictive of anthracycline-induced cardiotoxicity in children.. Pediatr Blood Cancer.

[58] Aminkeng F, Bhavsar AP, Visscher H, Rassekh SR, Li Y (2015). A coding variant in RARG confers susceptibility to anthracycline-induced cardiotoxicity in childhood cancer.. Nat Genet.

[59] Wojnowski L, Kulle B, Schirmer M, Schluter G, Schmidt A (2005). NAD(P)H oxidase and multidrug resistance protein genetic polymorphisms are associated with doxorubicin-induced cardiotoxicity.. Circulation.

[60] Chen SH, Pei D, Yang W, Cheng C, Jeha S (2010). Genetic variations in GRIA1 on chromosome 5q33 related to asparaginase hypersensitivity.. Clin Pharmacol Ther.

[61] Lopez-Santillan M, Iparraguirre L, Martin-Guerrero I, Gutierrez-Camino A, Garcia-Orad A (2017). Review of pharmacogenetics studies of L-asparaginase hypersensitivity in acute lymphoblastic leukemia points to variants in the GRIA1 gene.. Drug Metab Pers Ther.

[62] Lee JJ, Swain SM (2006). Peripheral neuropathy induced by microtubule-stabilizing agents.. J Clin Oncol.

[63] Zhang Y, Zolov SN, Chow CY, Slutsky SG, Richardson SC (2007). Loss of Vac14, a regulator of the signaling lipid phosphatidylinositol 3,5-bisphosphate, results in neurodegeneration in mice.. Proc Natl Acad Sci U S A.

[64] Sucheston-Campbell LE, Clay-Gilmour AI, Barlow WE, Budd GT, Stram DO (2018). Genome-wide meta-analyses identifies novel taxane-induced peripheral neuropathy-associated loci.. Pharmacogenet Genomics.

[65] Li W, Zhang H, Assaraf YG, Zhao K, Xu X (2016). Overcoming ABC transporter-mediated multidrug resistance: molecular mechanisms and novel therapeutic drug strategies.. Drug Resist Updat.

[66] Bruhn O, Cascorbi I (2014). Polymorphisms of the drug transporters ABCB1, ABCG2, ABCC2 and ABCC3 and their impact on drug bioavailability and clinical relevance.. Expert Opin Drug Metab Toxicol.

[67] Cascorbi I, Gerloff T, Johne A, Meisel C, Hoffmeyer S (2001). Frequency of single nucleotide polymorphisms in the P-glycoprotein drug transporter MDR1 gene in white subjects.. Clin Pharmacol Ther.

[68] Haenisch S, Zimmermann U, Dazert E, Wruck CJ, Dazert P (2007). Influence of polymorphisms of ABCB1 and ABCC2 on mRNA and protein expression in normal and cancerous kidney cortex.. Pharmacogenomics J.

[69] Plasschaert SL, Groninger E, Boezen M, Kema I, de Vries EG (2004). Influence of functional polymorphisms of the MDR1 gene on vincristine pharmacokinetics in childhood acute lymphoblastic leukemia.. Clin Pharmacol Ther.

[70] Zheng Q, Wu H, Yu Q, Kim DH, Lipton JH (2015). ABCB1 polymorphisms predict imatinib response in chronic myeloid leukemia patients: a systematic review and meta-analysis.. Pharmacogenomics J.

[71] Zu B, Li Y, Wang X, He D, Huang Z (2014). MDR1 gene polymorphisms and imatinib response in chronic myeloid leukemia: a meta-analysis.. Pharmacogenomics.

[72] Jeong H, Herskowitz I, Kroetz DL, Rine J (2007). Function-altering SNPs in the human multidrug transporter gene ABCB1 identified using a Saccharomyces-based assay.. PLoS Genet.

[73] Sakurai A, Tamura A, Onishi Y, Ishikawa T (2005). Genetic polymorphisms of ATP-binding cassette transporters ABCB1 and ABCG2: therapeutic implications.. Expert Opin Pharmacother.

[74] Scharenberg CW, Harkey MA, Torok-Storb B (2002). The ABCG2 transporter is an efficient Hoechst 33342 efflux pump and is preferentially expressed by immature human hematopoietic progenitors.. Blood.

[75] Jordanides NE, Jorgensen HG, Holyoake TL, Mountford JC (2006). Functional ABCG2 is overexpressed on primary CML CD34+ cells and is inhibited by imatinib mesylate.. Blood.

[76] Leonard GD, Fojo T, Bates SE (2003). The role of ABC transporters in clinical practice.. Oncologist.

[77] Litman T, Brangi M, Hudson E, Fetsch P, Abati A (2000). The multidrug-resistant phenotype associated with overexpression of the new ABC half-transporter, MXR (ABCG2).. J Cell Sci.

[78] Kim DH, Sriharsha L, Xu W, Kamel-Reid S, Liu X (2009). Clinical relevance of a pharmacogenetic approach using multiple candidate genes to predict response and resistance to imatinib therapy in chronic myeloid leukemia.. Clin Cancer Res.

[79] van der Veldt AA, Eechoute K, Gelderblom H, Gietema J, Guchelaar HJ (2011). Genetic polymorphisms associated with a prolonged progression-free survival in patients with metastatic renal cell cancer treated with sunitinib.. Clin Cancer Res.

[80] Rudin CM, Liu W, Desai A, Karrison T, Jiang X (2008). Pharmacogenomic and pharmacokinetic determinants of erlotinib toxicity.. J Clin Oncol.

[81] Sparreboom A, Loos WJ, Burger H, Sissung TM, Verweij J (2005). Effect of ABCG2 genotype on the oral bioavailability of topotecan.. Cancer Biol Ther.

[82] Takahashi N, Miura M, Scott SA, Kagaya H, Kameoka Y (2010). Influence of CYP3A5 and drug transporter polymorphisms on imatinib trough concentration and clinical response among patients with chronic phase chronic myeloid leukemia.. J Hum Genet.

[83] Dohse M, Scharenberg C, Shukla S, Robey RW, Volkmann T (2010). Comparison of ATP-binding cassette transporter interactions with the tyrosine kinase inhibitors imatinib, nilotinib, and dasatinib.. Drug Metab Dispos.

[84] Ripperger A, Benndorf RA (2016). The C421A (Q141K) polymorphism enhances the 3’-untranslated region (3’-UTR)-dependent regulation of ATP-binding cassette transporter ABCG2.. Biochem Pharmacol.

[85] Imai Y, Nakane M, Kage K, Tsukahara S, Ishikawa E (2002). C421A polymorphism in the human breast cancer resistance protein gene is associated with low expression of Q141K protein and low-level drug resistance.. Mol Cancer Ther.

[86] Poonkuzhali B, Lamba J, Strom S, Sparreboom A, Thummel K (2008). Association of breast cancer resistance protein/ABCG2 phenotypes and novel promoter and intron 1 single nucleotide polymorphisms.. Drug Metab Dispos.

[87] Cascorbi I, Werk AN (2017). Advances and challenges in hereditary cancer pharmacogenetics.. Expert Opin Drug Metab Toxicol.

[88] Rau T, Erney B, Gores R, Eschenhagen T, Beck J (2006). High-dose methotrexate in pediatric acute lymphoblastic leukemia: impact of ABCC2 polymorphisms on plasma concentrations.. Clin Pharmacol Ther.

[89] Au A, Baba AA, Azlan H, Norsa’adah B, Ankathil R (2014). Clinical impact of ABCC1 and ABCC2 genotypes and haplotypes in mediating imatinib resistance among chronic myeloid leukaemia patients.. J Clin Pharm Ther.

[90] Cuffe S, Azad AK, Qiu X, Qiu X, Brhane Y (2016). ABCC2 polymorphisms and survival in the Princess Margaret cohort study and the NCIC clinical trials group BR.24 trial of platinum-treated advanced stage non-small cell lung cancer patients.. Cancer Epidemiol.

[91] Kaehler M, Ruemenapp J, Gonnermann D, Nagel I, Bruhn O (2017). MicroRNA-212/ABCG2-axis contributes to development of imatinib-resistance in leukemic cells.. Oncotarget.

[92] Oberstadt MC, Bien-Moller S, Weitmann K, Herzog S, Hentschel K (2013). Epigenetic modulation of the drug resistance genes MGMT, ABCB1 and ABCG2 in glioblastoma multiforme.. BMC cancer.

[93] Bram EE, Stark M, Raz S, Assaraf YG (2009). Chemotherapeutic drug-induced ABCG2 promoter demethylation as a novel mechanism of acquired multidrug resistance.. Neoplasia.

[94] Reed K, Hembruff SL, Sprowl JA, Parissenti AM (2010). The temporal relationship between ABCB1 promoter hypomethylation, ABCB1 expression and acquisition of drug resistance.. Pharmacogenomics J.

[95] To KK, Zhan Z, Bates SE (2006). Aberrant promoter methylation of the ABCG2 gene in renal carcinoma.. Mol Cell Biol.

[96] Turner JG, Gump JL, Zhang C, Cook JM, Marchion D (2006). ABCG2 expression, function, and promoter methylation in human multiple myeloma.. Blood.

[97] Habano W, Kawamura K, Iizuka N, Terashima J, Sugai T (2015). Analysis of DNA methylation landscape reveals the roles of DNA methylation in the regulation of drug metabolizing enzymes.. Clin Epigenetics.

[98] Peng L, Zhong X (2015). Epigenetic regulation of drug metabolism and transport.. Acta Pharm Sin B.

[99] Zhao H, Wang D, Du W, Gu D, Yang R (2010). MicroRNA and leukemia: tiny molecule, great function.. Crit Rev Oncol Hematol.

[100] Shang Y, Zhang Z, Liu Z, Feng B, Ren G (2014). miR-508-5p regulates multidrug resistance of gastric cancer by targeting ABCB1 and ZNRD1.. Oncogene.

[101] Haenisch S, Laechelt S, Bruckmueller H, Werk A, Noack A (2011). Down-regulation of ATP-binding cassette C2 protein expression in HepG2 cells after rifampicin treatment is mediated by microRNA-379.. Mol Pharmacol.

[102] Werk AN, Bruckmueller H, Haenisch S, Cascorbi I (2014). Genetic variants may play an important role in mRNA-miRNA interaction: evidence for haplotype-dependent downregulation of ABCC2 (MRP2) by miRNA-379.. Pharmacogenet Genomics.

[103] To KK, Zhan Z, Litman T, Bates SE (2008). Regulation of ABCG2 expression at the 3’ untranslated region of its mRNA through modulation of transcript stability and protein translation by a putative microRNA in the S1 colon cancer cell line.. Mol Cell Biol.

[104] Bruhn O, Drerup K, Kaehler M, Haenisch S, Roder C (2016). Length variants of the ABCB1 3’-UTR and loss of miRNA binding sites: possible consequences in regulation and pharmacotherapy resistance.. Pharmacogenomics.

[105] He Y, Chevillet JR, Liu G, Kim TK, Wang K (2015). The effects of microRNA on the absorption, distribution, metabolism and excretion of drugs.. Br J Pharmacol.

[106] Zanger UM, Klein K, Kugler N, Petrikat T, Ryu CS (2018). Epigenetics and MicroRNAs in Pharmacogenetics.. Adv Pharmacol.

[107] Zheng T, Wang J, Chen X, Liu L (2010). Role of microRNA in anticancer drug resistance.. Int J Cancer.

[108] Polkowska M, Ekk-Cierniakowski P, Czepielewska E, Kozlowska-Wojciechowska M (2019). Efficacy and safety of BRAF inhibitors and anti-CTLA4 antibody in melanoma patients-real-world data.. Eur J Clin Pharmacol.

[109] Sharma P, Allison JP (2015). Immune checkpoint targeting in cancer therapy: toward combination strategies with curative potential.. Cell.

[110] Xu C, Chen YP, Du XJ, Liu JQ, Huang CL (2018). Comparative safety of immune checkpoint inhibitors in cancer: systematic review and network meta-analysis.. BMJ.

